# Inward Operation of Sodium-Bicarbonate Cotransporter 1 Promotes Astrocytic Na^+^ Loading and Loss of ATP in Mouse Neocortex during Brief Chemical Ischemia

**DOI:** 10.3390/cells12232675

**Published:** 2023-11-21

**Authors:** Katharina Everaerts, Pawan Thapaliya, Nils Pape, Simone Durry, Sara Eitelmann, Eleni Roussa, Ghanim Ullah, Christine R. Rose

**Affiliations:** 1 Institute of Neurobiology, Heinrich Heine University Düsseldorf, Universitätsstraße 1, D-40225 Düsseldorf, Germany; katharina.everaerts@hhu.de (K.E.); nils.pape@hhu.de (N.P.); simone.durry@hhu.de (S.D.); sara.eitelmann@hhu.de (S.E.); 2 Department of Physics, University of South Florida, Tampa, FL 33620, USA; pkthapaliya@usf.edu (P.T.); gullah@usf.edu (G.U.); 3 Institute of Anatomy and Cell Biology, Department of Molecular Embryology, Faculty of Medicine, Albert-Ludwigs-Universität Freiburg, Albertstrasse 17, D-79104 Freiburg, Germany; eleni.roussa@anat.uni-freiburg.de

**Keywords:** astrocyte, sodium, pH, neocortex, ischemia, ATP, imaging, modeling, excitotoxicity

## Abstract

Ischemic conditions cause an increase in the sodium concentration of astrocytes, driving the breakdown of ionic homeostasis and exacerbating cellular damage. Astrocytes express high levels of the electrogenic sodium-bicarbonate cotransporter1 (NBCe1), which couples intracellular Na^+^ homeostasis to regulation of pH and operates close to its reversal potential under physiological conditions. Here, we analyzed its mode of operation during transient energy deprivation via imaging astrocytic pH, Na^+^, and ATP in organotypic slice cultures of the mouse neocortex, complemented with patch-clamp and ion-selective microelectrode recordings and computational modeling. We found that a 2 min period of metabolic failure resulted in a transient acidosis accompanied by a Na^+^ increase in astrocytes. Inhibition of NBCe1 increased the acidosis while decreasing the Na^+^ load. Similar results were obtained when comparing ion changes in wild-type and *Nbce1*-deficient mice. Mathematical modeling replicated these findings and further predicted that NBCe1 activation contributes to the loss of cellular ATP under ischemic conditions, a result confirmed experimentally using FRET-based imaging of ATP. Altogether, our data demonstrate that transient energy failure stimulates the inward operation of NBCe1 in astrocytes. This causes a significant amelioration of ischemia-induced astrocytic acidification, albeit at the expense of increased Na^+^ influx and a decline in cellular ATP.

## 1. Introduction

Astrocytes are central to brain function. Long-established roles of astrocytes include the uptake of K^+^ from the extracellular space (ECS) and the regulation of extracellular K^+^ homeostasis [1]. Uptake of K^+^ is mainly mediated by the astrocytic Na^+^/K^+^-ATPase (NKA), a major consumer of cellular ATP [2,3]. In addition to its role in extracellular K^+^ homeostasis, the NKA is responsible for the export of Na^+^ and the maintenance of a low intracellular Na^+^ concentration ([Na^+^]_i_) [4]. Astrocytes, moreover, play a key role in the uptake and regulation of the neurotransmitter glutamate, which is mediated by the Na^+^-dependent high-affinity transporters GLAST and GLT-1 (EAAT1 and EAAT2, respectively) [5,6]. Both processes rely on an intact energy metabolism and ATP production to enable NKA activity and the maintenance of a strong inward gradient for Na^+^.

In the core region of an ischemic stroke, intracellular ATP levels fall, and astrocytes suffer from a breakdown of ionic homeostasis, including an increase in their [Na^+^]_i_ [7,8,9]. Prominent, albeit more transient, changes in astrocytic [Na^+^]_i_ are observed in the ischemic penumbra during the passage of a wave of spreading depolarization [9,10,11]. Earlier work has shown that an increase in [Na^+^]_i_ results in a reduced capacity for glutamate uptake or even in the reversal of glutamate transporters and reduced glial uptake of K^+^ [12,13,14]. Moreover, Na^+^ loading promotes the reversal of the plasma membrane Na^+^/Ca^2+^-exchanger (NCX), thereby contributing to the deleterious Ca^2+^ loading of astrocytes [10,15,16,17,18].

Another Na^+^-dependent transporter whose driving force is affected by changes in astrocytic [Na^+^]_i_ is the electrogenic sodium-bicarbonate cotransporter 1 (NBCe1; *SLC4A4*) [19]. Outward transport of HCO_3_^−^ via the NBCe1 is a major mechanism for regulation of intracellular pH (pH_i_) in astrocytes when pH_i_ is above 6.8 [20,21,22]. Depending on the cellular membrane potential and the respective ion concentrations, it can mediate either the influx of Na^+^ and HCO_3_^−^ (forward/inward mode) or their efflux (reverse/outward mode). While the forward operation of NBCe1 is activated, for example, in response to neuronal activity and an increase in the extracellular K^+^ concentration ([K^+^]_o_) [23,24,25,26], its reverse operation can be induced by a decrease in extracellular HCO_3_^−^ ([HCO_3_^−^]_o_) or by inhibition of the NKA and the concomitant increase in astrocytic [Na^+^]_i_ [27,28]. In addition to ionic driving forces, NBCe1 is modulated via several intracellular signaling pathways that influence its activity [29,30].

The mode of operation of NBCe1 in ischemic conditions, however, is unclear. In the ischemic brain, cells not only undergo a membrane depolarization and an increase in [Na^+^]_i_, but also a long-lasting acidification [7,8,9,11,31,32], and all of these processes will strongly affect the driving forces for NBCe1. It is therefore unclear whether NBCe1 activity promotes (forward mode) or dampens (reverse mode) astrocytic Na^+^ loading. Furthermore, it is unclear if (and how) NBCe1 activity contributes to astrocytic pH_i_ changes in ischemic conditions.

To address these questions, we performed fluorescence imaging with the chemical indicator dyes SBFI and BCECF to measure changes in astrocytic [Na^+^]_i_ and pH_i_, respectively, in organotypic slice cultures of the murine neocortex. Astrocytic ATP levels were analyzed using the genetically encoded fluorescent sensor ATeam1.03^YEMK^ [33]. The involvement of NBCe1 activity was probed using pharmacological tools and by comparing slice preparations derived from wild-type and *Nbce1*-deficient animals. Experimental results allowed for a comprehensive mathematical simulation of ion fluxes and NBCe1 activity during metabolic failure and enabled the prediction of NBCe1-related ATP consumption in astrocytes. Our experimental results and computational modeling demonstrate that a 2 min transient energy failure stimulates inwardly directed NBCe1 activity in astrocytes. This increases Na^+^ influx and astrocytic ATP consumption while at the same time dampening their acidification.

## 2. Materials and Methods

### 2.1. Preparation of Organotypic Slice Cultures

In the present study, we used wild-type mice as well as transgenic, *Nbce1*-deficient mice (NBCe1 KO [34]; originally received from Dr. Gary E. Shull, UC College of Medicine, University of Cincinnati, Cincinnati, OH, USA). For the preparation of organotypic hippocampal slice cultures, mice (*Mus musculus*, Balb/C; both sexes) ranging from postnatal days (P) 6–9 were used, on which most experiments were performed. In addition, neonatal NBCe1 KO mice were used from P5 to 6 [34]. C57BL/6N mice (both sexes) served as wild-type controls for these animals.

Acute slices of 250 µm thickness were prepared using the methods previously published [35]. Briefly, mice were quickly decapitated, and brains were immediately placed in ice-cold standard artificial cerebrospinal fluid (aCSF) containing (in mM): 130 NaCl, 2.5 KCl, 2 CaCl_2_, 1 MgCl_2_, 1.25 NaH_2_PO_4_, 26 NaHCO_3_, and 10 glucose, which was bubbled with 5% CO_2_/95% O_2_, resulting in a pH of 7.35–7.4. Brains were separated into hemispheres and cut parasagittally using a vibratome (HM 650 V; Thermo Fisher Scientific, Waltham, MA, USA). Acute slices were cultured following a procedure by Stoppini [36] and as described in more detail recently [37]. Slice cultures were kept in an incubator at 36 °C at the interface between humidified carbogen (5% CO_2_/95% O_2_) and culture medium containing minimum essential medium (MEM; M7M278), 20% heat-inactivated horse serum (Origin, Brazil; Thermo Fisher Scientific, Waltham, MA, USA), 1 mM of L-glutamine, 0.01 mg mL^−1^ of insulin, 14.5 mM of NaCl, 2 mM of MgSO_4_, 1.44 mM of CaCl_2_, 0.00125% ascorbic acid, and 13 mM of D-glucose. The medium was replaced every 3 days.

If not stated otherwise, experiments were performed on organotypic slices cultured between 10 and 21 days. For astrocyte identification, slices were exposed to 0.5–1 µM of sulforhodamine 101 (SR101) in aCSF for 30–60 min in the incubator. Experiments were carried out at room temperature (21 ± 1 °C). Throughout experiments, slices were perfused with aCSF containing 0.5 µM of tetrodotoxin (TTX, Biotrend, Cologne, Germany) to block action potential discharges, typical for organotypic slice preparations [38]. Pharmacological substances were diluted in aCSF and bath-applied via the perfusion system for 15 min before the beginning of and throughout the recordings. Brief chemical ischemia was induced via a 2 min bath application of glucose-free aCSF containing sodium azide (NaN_3_, 5 mM; inhibitor of cytochrome C oxidase and hence mitochondrial respiration) as well as 2-deoxyglucose (2-DG, 2 mM; inhibitor of hexokinase and thus glycolysis). If not stated otherwise, chemicals were purchased from Sigma-Aldrich (Munich, Germany).

### 2.2. Imaging of Intracellular pH and Na^+^

For the determination of changes in pH_i_ or [Na^+^]_i_, we performed wide-field imaging using an epifluorescence microscope (Nikon Eclipse FN 1, Nikon Europe, Düsseldorf, Germany), equipped with a Fluor 40×/0.8 NA water immersion objective (Nikon) coupled to a Poly-V monochromator (Thermo Scientific/FEI, Planegg, Germany). After staining for astrocytes with SR101, organotypic slice cultures were bolus-loaded with fluorescent indicators for the respective ion, as reported before [35].

Changes in pH_i_ were determined by loading the slices with BCECF (BCECF-AM, 125 µM, A.G. Scientific, San Diego, CA, USA). Standard dual excitation ratiometric imaging of BCECF was performed by alternating excitation at 458 (isosbestic wavelength) and 488 nm (pH-sensitive wavelength) and recording fluorescence emission ranging between 518 and 563 nm using a 518 beam splitter and a 537/26 band pass filter.

Changes in [Na^+^]_i_ were determined using SBFI (SBFI-AM, 116.7 µM, ION Biosciences, San Marcos, TX, USA). SBFI was excited at 400 nm, and its emission was detected above ~430 nm using a 409 beam splitter and a band pass 510/84 nm emission filter [10]. Images were obtained at 0.5–1 Hz with an ORCA FLASH 4.0LT camera (Hamamatsu Photonics, Herrsching, Germany). Emission was collected from regions of interest (ROIs) representing the cell bodies of SR101-positive astrocytes and analyzed using OriginPro 2019 (OriginLab Corporation, Northampton, MA, USA). Fluorescence emission from individual ROIs was background-corrected as reported earlier [35]. Afterwards, the fluorescence ratio (F_458_/F_488_) was calculated for BCECF.

Changes in SBFI emission and BCECF ratio were converted into mM (Na^+^) and pH units, respectively, using established in situ calibration procedures [10,39,40]. In brief, for the calibration of SBFI, SBFI -loaded slices were perfused with calibration salines containing [Na^+^] ranging from 0 to 150 mM, as well as the NKA inhibitor ouabain (100 µM), the Na^+^/H^+^ exchanger monensin (10 µM), and the Na^+^ ionophore gramicidin (3 µM) to equilibrate intra- and extracellular Na^+^. Calibration was started by perfusing the slices with nominally Na^+^-free calibration saline. Subsequently, slices were exposed to calibration solutions containing 5–150 mM Na^+^ before going back to nominally Na^+^-free calibration saline. Detected changes in SBFI fluorescence were normalized to the initial baseline in nominally Na^+^-free calibration saline and plotted against the respective Na^+^ concentration. The data followed a Michaelis-Menten relationship (R^2^ = 0.975), revealing an apparent K_D_ of about 32 mM (not illustrated). This calibration curve enabled a calculation of changes in astrocytic [Na^+^]_i_, assuming a baseline [Na^+^]_i_ of 12.1 mM as determined for neocortical astrocytes in a recent study from our laboratory (baseline [Na^+^]_i_: 12.1 ± 0.5 mM [15]).

For calibration of BCECF, we used calibration salines from pH 5.5 to 8.5 (0.5 increments) containing the K^+^/H^+^ exchanger nigericin (10 µM) to equilibrate intra- and extracellular pH. Calibration was started by perfusing BCECF-loaded slices with a calibration saline at pH 5.5 and then switching to other calibration salines. Mean changes in the astrocytic BCECF ratio (F_458_/F_488_) were recorded, normalized to pH 5.5, and then plotted against the respective pH. A linear fit between pH 6.5 and 8.0 (R^2^ = 0.982; not illustrated) enabled the calculation of changes in astrocytic pH_i_, assuming a baseline pH_i_ of 7.33 as determined for neocortical astrocytes in a recent study from our laboratory (baseline pH_i_: 7.33 ± 0.04 [41]). The data were analyzed further offline using OriginPro 2019.

Of note, our in situ calibrations allow a reliable and reproducible calculation of absolute changes in ion concentrations ([Na^+^]_i_, pH_i_) at a given rig at which experiments are performed. However, initial tissue swelling and movement upon perfusion with calibration salines prevent a direct readout of cellular baseline ion concentrations in physiological saline. Determination of the latter thus requires alternative strategies and experimental approaches as described before (e.g., [15,41]). Importantly, the range of the near-linear behavior of both dyes (SBFI and BCECF) fully covers and even exceeds the range ± S.D. of baseline [Na^+^]_i_ (12.1 ± 0.5 mM) and pH_i_ (7.33 ± 0.04) determined earlier (e.g., [15,41]). This means that even if baseline ion concentrations differ between different cells to some extent, this will not distort the calculation made of the absolute changes in ion concentrations presented throughout the present manuscript.

### 2.3. FRET-Based Imaging of Intracellular ATP

For the determination of changes in astrocytic ATP levels in organotypic slice cultures, we employed the genetically encoded, fluorescence resonance energy transfer (FRET)-based sensor ATeam1.03^YEMK^ (ATeam) [33]. To this end, 0.5 µL of a vector (AAV 2/5) carrying the coding sequence for ATeam under the transcriptional control of the human glial fibrillary acid protein (*hGFAP*) promoter fragment ABC1D was applied to the top of a cultured slice at 1–3 days in vitro as described before [42]. After transduction, slices were maintained in the incubator for at least 10 more days before being used for experiments.

Slices expressing ATeam were imaged using an upright microscope (Nikon Eclipse FN-I, Nikon GmbH Europe, Düsseldorf, Germany) equipped with an Achroplan 40×/0.8 NA water immersion objective (Nikon). ATeam was excited using a Poly-V monochromator (Thermo Fisher Scientific/FEI, Planegg, Germany) at 434 nm, and images were taken at 0.5 Hz with a CMOS camera (Orca 4 LT Plus, Hamamatsu Photonics, Herrsching, Germany). Fluorescence emission was split at ~500 nm using a 505 beam splitter (WVIEW GEMINI optic system; Hamamatsu Photonics, Herrsching, Germany) onto two band pass filters (483/32 nm, imaging of the enhanced cyan fluorescent protein (*eCFP*)/donor; and 542/27 nm, imaging of Venus/acceptor). After background correction, the fluorescence ratio (Venus/eCFP) was calculated for individual ROIs, representing the cell bodies of astrocytes. Subsequent analysis was performed using OriginPro 2021 software (OriginLab Corporation, Northampton, MA, USA). Normalized changes in the Venus/eCFP fluorescence ratio are given as a percentage change thereof (ΔATeam ratio (%)).

### 2.4. Measurement of Extracellular K^+^, pH, and Na^+^

Measurements of extracellular ion shifts were obtained by employing double-barreled ion-sensitive microelectrodes as described before (e.g., [41,43]). In brief, two thin-walled borosilicate glass capillaries with filament were glued and pulled out together. The tip of one capillary was silanized via exposure to vaporized hexamethyldisilazane (Fluka, Buchs, Switzerland) and filled with a liquid neutral ion carrier based on valinomycin for measurement of [K^+^]_o_ (Ionophore I, Cocktail B, Fluka, Buchs, Switzerland), Hydrogen Ionophore I for pH (Cocktail A, 95291, Merck, Darmstadt, Germany), or ETH 157 for [Na^+^]_o_ (Ionophore II, Cocktail A, Fluka, Buchs, Switzerland). Afterwards, the capillary was backfilled with 100 mM NaCl or 100 mM KCl. The reference electrode was filled with 150 mM NaCl/1 mM HEPES (titrated to pH 7.0 with NaOH).

Electrodes were calibrated directly before and after each individual experiment. Calibration of K^+^-sensitive electrodes was performed in HEPES-based saline containing a total of 150 mM NaCl and KCl, in which KCl was 0–10 mM and NaCl adjusted accordingly to maintain osmolarity. For calibration of Na^+^-sensitive electrodes, solutions with Na^+^ concentrations ranging from 70 to 160 mM, to which N-methyl-D-glucamine chloride (NMDG-Cl) was added to adjust osmolarity (326 ± 5 mOsm l^−1^), were used. pH-sensitive microelectrodes were calibrated with salines titrated to a pH of 7.0 or 7.6, containing (in mM): 144.25 NaCl (pH 7.0)/108.48 NaCl (pH 7.6), 2.5 KCl, 1.25 NaH_2_PO_4_, and 12 NaHCO_3_ (pH 7.0)/47.77 NaHCO_3_ (pH 7.6), bubbled with carbogen. The data were processed in OriginPro 2019.

### 2.5. Patch-Clamp Recordings

Changes in astrocytic membrane potential were monitored using cell-attached patch-clamp recordings. These were carried out at an upright microscope (E600FN, Nikon, Tokyo, Japan), which was equipped with infrared differential interference contrast optics. The latter included a 60× water immersion objective (Fluor 60×/1.00 W, DIC H/N2, ∞/0 WD 2.0, Nikon) and an infrared video camera (XC ST70CE, Hamamatsu Photonics, Herrsching, Germany). Patch pipettes with a resistance of 3.5–4.0 MΩ (when filled with external solution) were pulled from borosilicate glass capillaries (GB150(F) 8P, Science Products, Hofheim am Taunus, Germany) using a vertical puller (PC-10 Puller, Narishige International, London, UK). Electrophysiological measurements were performed using an EPC10 amplifier and “PatchMaster” software (Harvard Bioscience/HEKA Elektronik, Lambrecht, Germany). Recordings in cell-attached configuration were conducted as reported recently [41]. In brief, pipettes were filled with standard aCSF, and the offset potential was corrected. After achieving a seal higher than 1 GΩ, cells were recorded for 30–45 min to ensure reliable measurement of the membrane potential [44]. The data were analyzed offline using OriginPro 2021.

### 2.6. Modeling NBCe1 Activity

We build on our previous work, where we modeled dynamic changes in astrocytic Na^+^, K^+^, Cl^−^, and Ca^2+^ concentrations [45,46] by incorporating the main pathways regulating intra- and extracellular pH (pH_i_ and pH_o_, respectively). The equations for intra- and extracellular Na^+^ concentrations ([Na^+^]_i_ and [Na^+^]_o_, respectively) are modified accordingly. The rate equations for various state variables and related fluxes are detailed in Appendix A. A schematic of the model is shown in the results section (see Figure 4). Here, we only discuss equations related to pH.

pH_o_ and astrocyte pH_i_ are controlled with fluxes through NBCe1 (J_NBCe1_), sodium/proton exchanger (J_NHE_), and diffusion between the ECS and bath solution, that is

(1) $$\frac{dpH_{o}}{dt}=\frac{1}{VR_{sa}\beta }(-J_{NHE}-J_{NBCe1})+diff(pH_{bath}-pH_{o})$$

(2) $$\begin{array}{rr} \frac{dpH_{i}}{dt} & =\frac{J_{NHE}+J_{NBCe1}}{\beta }, \end{array}$$

where diff is the rate at which pH_o_ equilibrates with the bath solution (pH_bath_) and VR_sa_ is the ECS to astrocytic volume ratio.

$\beta =\beta_{i/o}+\frac{{2.3\left[HCO_{3}^{-}\right]}_{i/o}}{[p{H_{rest}]}_{i/o}}$ is the total pH buffering capacity, where $\beta_{i/o}$= $\Delta {\left[HCO_{3}^{-}\right]}_{i/o}/\Delta pH$ is the intrinsic buffering capacity. [HCO_3_^−^]_i/o_ is the intra- and extracellular bicarbonate concentration, respectively, and [pH_rest_]_i/o_ is the resting pH of the intra- (7.33) and extracellular (7.35) regions in our experiments. ΔpH is the maximum observed change in pH with respect to the resting state inside or outside the cell. In our experiments, the highest change occurred in pH_i_ (that is, the maximum change in pH_o_ is lower), thus we used the maximum observed change in pH_i_ as ΔpH. We observed that the peak change in [HCO_3_^−^]_i_, [HCO_3_^−^]_o_, and pH_i_ with respect to their resting values was 12 mM, 5 mM, and 0.48 pH units, respectively. All these considerations led to intra- and extracellular intrinsic buffering powers of 25 mM/pH and 10.5 mM/pH, respectively. Notice that β_i/o_ is the slope of the [HCO_3_^−^] versus pH curve. Thus, our estimate of β_i/o_ is an average approximation as we look at the maximum changes in pH and [HCO_3_^−^] in our experiments. To measure the value β_i/o_ as a continuous function of pH, one would have to measure [HCO_3_^−^] at multiple pH values and find the slope of the curve at different pH values.

The second expression in β represents the buffering power of the intra- and extracellular regions in an open system at a steady-state pH due to CO_2_. Our model assumes fixed CO_2_, so carbonic acid (H_2_CO_3_) concentration does not change. We remark that replacing [pH_rest_]_i/o_ in the second term in β with pH unit (i.e., removing [pH_rest_]_i/o_ from the equation) does not change our results significantly. Thus, we leave the equation unperturbed. Furthermore, the effect of surrounding cells other than the astrocyte modeled on pH_o_ is incorporated in the diffusion term. However, some pathways regulating pH_i_, including H^+^ and HCO_3_^−^ leaks, background acid loading, and CO_2_ dissociation and respiration, are not included, as the goal was to develop a simple model capable of reproducing our observations. However, as more data on the pH regulation mechanism of astrocytes emerge, incorporating these and other pH-regulating pathways in the model will be crucial, which is the subject of our future research.

The equations for J_NBCe1_ and J_NHE_ are similar to those used in [47]. That is,

(3) $$\begin{array}{r} J_{NBCe1}=G_{NBCe1}(v_{i}-E_{NBCe1}), \end{array}$$

where E_NBCe1_, *v*_i_, and G_NBCe1_ are the reversal potential for Na^+^ and HCO_3_^−^ flux through NBCe1, the membrane potential of astrocytes, and the whole-cell conductance of NBCe1, respectively. E_NBCe1_ is calculated using the Nernst equation.

(4) $$E_{NBCe1}=\frac{V_{T}}{z_{NBCe1}}\ln\left(\frac{[N{a^{+}]}_{o}}{{\left[HCO_{3}^{-}\right]}_{i}^{2}}\frac{{\left[HCO_{3}^{-}\right]}_{o}^{2}}{{\left[Na^{+}\right]}_{i}}\right).$$

$V_{T}=\frac{RT}{F}$, where R, T, and F represent the gas constant, temperature, and Faraday’s constant and Z_NBCe1_ represents the net charge transported. [HCO_3_^−^]_i_ is the intracellular bicarbonate concentration. J_NHE_ is given as

(5) $$J_{NHE}=G_{NHE}(v_{i}-E_{NHE}),$$

where G_NHE_ is the whole-cell conductance of NHE and E_NHE_ is its reversal potential.

(6) $$E_{NHE}=\frac{V_{T}}{z_{NHE}}\ln\left(\frac{[{Na^{+}]}_{i}{\left[H^{+}\right]}_{o}}{{\left[Na^{+}\right]}_{o}[{H^{+}]}_{i}}\right).$$

[H^+^]_o_ and [H^+^]_i_ represent extra- and intracellular hydrogen concentrations, respectively. Z_NBCe1_ is the net charge transported using NHE.

[HCO_3_^−^]_o_ is given using the Henderson-Hasselbalch equation [48],

(7) $${\left[HCO_{3}^{-}\right]}_{o}=10^{\left(pH_{o}-pK_{a}\right)}[CO_{2\left(aq\right)}],$$

where pK_a_ is the negative logarithm (base = 10) of the acid dissociation constant of carbonic acid, and [CO_2(aq)_] is the product of solubility (s) in aqueous solution or water and partial pressure of carbon dioxide (P_CO2_). Similarly, [HCO_3_^−^]_i_ is given as

(8) $${\left[HCO_{3}^{-}\right]}_{i}=10^{\left(pH_{o}-pH_{i}\right)}{\left[HCO_{3}^{-}\right]}_{o}.$$

[H^+^]_o_ and [H^+^]_i_ are calculated using the Kassirer–Bleich approximation [48],

(9) $${\left[H^{+}\right]}_{i/o}=\frac{sK_{h}P_{CO_{2}}}{{\left[HCO_{3}^{-}\right]}_{i/o}},$$

where K_h_ is the dissociation constant of carbonic acid.

The equations for NKA are modified to simulate 2 min chemical ischemia as explained in Appendix A.

### 2.7. Numerical Methods

The rate equations are solved in Fortran 90 using the Euler method with a time step of 0.1 µs. The system of equations is allowed to reach a steady state before imposing chemical ischemia. The data are visualized using MATLAB (MATLAB Version: 9.14.0.2286388 (R2023a) Update 3, The MathWorks Inc., Natick, MA, USA). The codes reproducing the main results are available from the authors upon request.

### 2.8. Data Analysis and Statistics

Each series of experiments on tissue slices was prepared from at least three different animals; “n” represents the number of cells analyzed and “N” the number of individual experiments/slices. In the results section, numbers for individual experiments are stated as follows: number of cells investigated/number of different slices/number of different animals. Power analysis was conducted using G*Power 3.1.9.6 [49]. The effect size was calculated as the difference of means divided by the standard deviation of the control group. For a significance level of 0.001, it revealed a minimum power of 0.8. Results are given as the mean ± standard deviation (SD). Data are illustrated in box plots indicating the mean (black square), interquartile range (box), median (middle line), and SD (whiskers). Additionally, all individual data points are shown underneath box plots as dots. The data were statistically analyzed using OriginPro 2019. The normality of the data was assessed using a Shapiro-Wilk test. In the case of a normal distribution, the data were statistically analyzed using either a Student’s *t*-test (“*t*-test”) or a one-way ANOVA (“ANOVA”), followed by a post hoc Bonferroni test. Otherwise, statistical analysis was conducted using a U-test (Mann-Whitney, “MWU”). *p*-values below 0.05 were considered to indicate a significant difference. The following symbols are used to illustrate the results of statistical tests in the figures (*p* represents the error probability): * *p* < 0.05, ** *p* < 0.01, and *** *p* < 0.001.

## 3. Results

### 3.1. Probing NBCe1 Activity in Organotypic Slice Cultures

The goal of the present study was to reveal the mode of operation of NBCe1 in astrocytes of the mouse neocortex during short periods of energy failure and to analyze its effects on ischemia-induced changes in astrocytic pH_i_ and [Na^+^]_i_. To test whether NBCe1 is functionally active in organotypic slice cultures and can be addressed pharmacologically, we first probed for a Depolarization-Induced *A*lkalinization (“DIA”) of astrocytes, which is based on stimulation of inward NBCe1 activity [50,51].

**Figure 1:**
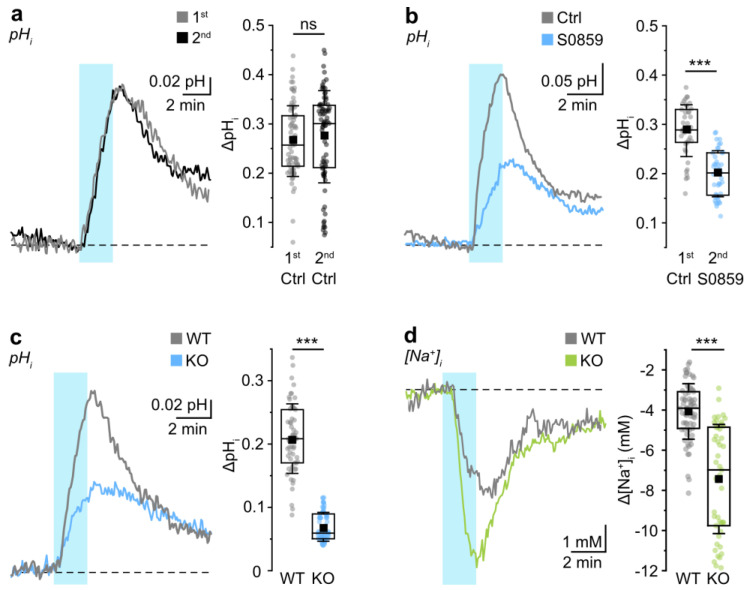
K^+^-induced changes in astrocytic pH_i_ and [Na^+^]_i_. The left panels of (**a**–**d**) show representative traces from single experiments; the right panels show data for all the experiments. (**a**) Left: Change in pH_i_ evoked by two consecutive increases in [K^+^]_o_ for 2 min each. (**b**) Left: Change in pH_i_ evoked by two consecutive increases in [K^+^]_o_ for 2 min in control conditions (1st application, Ctrl) and in the presence of the NBC inhibitor S0859 (2nd application). (**c**) Left: Change in pH_i_ evoked by an increase in [K^+^]_o_ for 2 min in WT and in NBCe1 KO animals. (**d**) Left: Change in astrocytic [Na^+^]_i_ evoked by an increase in [K^+^]_o_ for 2 min in WT and in NBCe1 KO animals. Periods of increased [K^+^]_o_ are indicated with the light blue areas; upward traces represent alkalinizations. Dashed lines indicate baseline levels. Box plots on the right summarize the results from these experiments and show individual data points at maximum change (dots), means (squares), interquartile ranges (boxes), medians (lines), and SD (whiskers). ns: non-significant; *** *p* < 0.001.

To depolarize astrocytes, we elevated the K^+^ concentration of the aCSF from 2.5 to 10 mM for 2 min. Recordings with K^+^-sensitive microelectrodes showed that this resulted in a transient increase in the [K^+^]_o_ within the slice preparation from 2.5 to 8.6 mM (3/3/3; not illustrated). Raising [K^+^]_o_ indeed resulted in an alkalinization of astrocytes, amounting to on average 0.26 ± 0.07 pH units (81/7/4) (Figure 1a). The amplitude of this alkalinization remained stable upon a second elevation of [K^+^]_o_, performed 30 min after the first one (amplitude at second application: 0.27 ± 0.09 pH units; 81/7/4; MWU, *p* = 0.221) (Figure 1a).

To probe for the involvement of sodium-bicarbonate co-transport (NBC) in this alkalinization, we applied S0859 (30 µM), an inhibitor of the NBC transporter family [52]. At an assumed average baseline pH_i_ of 7.33 ([41], see methods), we found that pH_i_ was 0.01 ± 0.10 pH units lower after the addition of S0859 than in the control. Thus, S0859 did not alter pH_i_ significantly compared to the control prior to its addition (control: 7.33 ± 0.04, 8/5/4; S0589: 7.32 ± 0.09, 59/5/5; *t*-test, *p* = 0.592). However, in the presence of S0859, the K^+^-induced astrocytic alkalinization was reduced from 0.29 ± 0.05 pH units (first application, control) to 0.20 ± 0.05 pH units (second application with blocker) (42/5/4; ANOVA, *** *p* < 0.001) (Figure 1b).

We next analyzed astrocytes in organotypic slice preparations derived from NBCe1 KO mice [34], for which slices from C57BL/6N mice served as wild-type controls (WT). In WT animals, the 2 min elevation in [K^+^]_o_ caused an alkalinization of 0.21 ± 0.05 pH units (57/6/3) (Figure 1c). Astrocytes in slices of NBCe1 KO displayed a significantly reduced alkalinization of 0.07 ± 0.02 pH units (75/5/3; MWU, *** *p* > 0.001) (Figure 1c).

Finally, we studied K^+^-induced changes in astrocytic [Na^+^]_i_ in both WT and NBCe1 KO mice. The elevation of [K^+^]_o_ caused a decrease in [Na^+^]_i_ in WT mice by 4.1 ± 1.4 mM (67/5/3) (Figure 1d). In NBCe1 KO, this decrease was significantly larger (7.4 ± 2.7 mM; 50/5/4; MWU, *** *p* < 0.001) (Figure 1d), indicating reduced Na^+^ influx under these conditions.

In summary, our data demonstrate functional NBCe1 activity in astrocytes from organotypic brain slices. They show that a moderate, transient elevation of [K^+^]_o_ induces a transient astrocytic alkalinization, which is significantly reduced by the NBC inhibitor S0859. Moreover, the amplitude of this alkalinization is significantly reduced in animals deficient in *Nbce1*. The latter also show a larger drop in [Na^+^]_i_ in response to elevation of [K^+^]_o_, suggesting less Na^+^ influx than in WT animals. These effects are all in line with a stimulation of inward NBCe1 in response to elevation of [K^+^]_o_ and demonstrate that this transporter is functionally active in astrocytes of organotypic slices of mouse neocortex.

### 3.2. Role of NBCe1 Activity in Astrocytic Ion Changes during Brief Chemical Ischemia

After demonstrating functional activity of NBCe1 in astrocytes of organotypic slice cultures, we investigated its role in the generation of changes in astrocytic pH_i_ and [Na^+^]_i_ evoked by short periods of energy deprivation. The latter was induced by perfusing slices for 2 min with a glucose-free saline containing 5 mM of NaN_3_ and 2 mM of 2-DG to block cellular ATP production by blocking mitochondrial respiration and glycolysis, respectively (“chemical ischemia”) [10,41,53].

**Figure 2:**
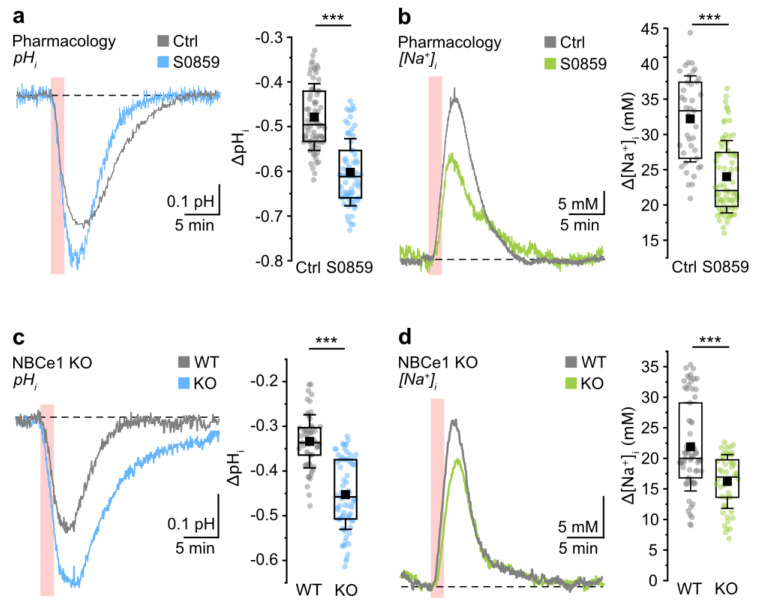
Role of NBCe1 in ischemia-induced changes in astrocytic pH_i_ and [Na^+^]_i_. Data are presented the same as in Figure 1. (**a**) Change in pH_i_ evoked by chemical ischemia for 2 min in control and with addition of S0859. (**b**) Change in [Na^+^]_i_ evoked by chemical ischemia in control (Ctrl) and with addition of S0859. (**c**) Change in pH_i_ evoked by chemical ischemia for 2 min in slices derived from wild-type mice (WT) and from NBCe1 KO. (**d**) Left: Change in [Na^+^]_i_ in wild-type and NBCe1 KO. Periods of chemical ischemia are indicated with the light-red areas; downward traces represent acidification. Dashed lines indicate baseline levels. Box plots on the right summarize the results from these experiments and show individual data points (dots), means (squares), interquartile ranges (boxes), medians (lines), and SD (whiskers). *** *p* < 0.001.

As expected from earlier work [7,41,54,55], brief chemical ischemia resulted in an acidification of astrocytes, decreasing their pH_i_ by 0.48 ± 0.07 pH units (79/5/3) (Figure 2a). With ATP production from glucose metabolism and oxidative respiration blocked, a likely mechanism contributing to astrocyte acidosis is e.g. the activation of glutamate transporters and the uptake of protons in conjunction with glutamate [56]. The peak acidification was reached within 183 ± 26 s, after which astrocytes slowly recovered back to their initial pH_i_ (full width at half maximum (FWHM): 399 ± 70 s). Inhibition of NBC via bath application of 30 µM of S0859 resulted in a significantly larger acidification by 0.60 ± 0.08 pH units upon chemical ischemia (70/4/3; MWU, *** *p* < 0.001) (Figure 2a). Notably, only half of the cells recovered to their initial baseline pH_i_, indicating a compromised ability for the export of acid equivalents under these conditions.

In addition to the acidification, chemical ischemia evoked a transient increase in astrocytic [Na^+^]_i_ by 33.8 ± 8.7 mM, from which cells recovered within about 15 min (39/4/4) (Figure 2b). Assuming an average baseline [Na^+^]_i_ of 12.1 mM in neocortical astrocytes ([15], see methods), we found that addition of S0859 did not alter [Na^+^]_i_ significantly (S0589: 12.45 ± 0.97 mM (31/3/3); *t*-test, *p* = 0.118). Upon application of S0859, the peak amplitude of the chemical-ischemia-induced [Na^+^]_i_ increase was significantly smaller than in control, amounting to 22.1 ± 7.3 mM (76/6/3; MWU, *** *p* < 0.001) (Figure 2b). As in the control, and opposed to the pH changes induced, cells recovered fully from the [Na^+^]_i_ increase during pharmacological inhibition of NBCe1.

Next, we analyzed the effects of the 2 min chemical ischemia in organotypic slices from NBCe1 KO animals. Again, astrocytes in organotypic slices prepared from C57BL/6N wild-type mice were used as controls (see methods and above). We found that in the control, astrocytes acidified by 0.33 ± 0.06 pH units, from which cells fully recovered (FWHM: 410 ± 81 s) (57/6/3) (Figure 2c). Cells from *Nbce1*-deficient mice showed a significantly larger acidification of 0.45 ± 0.08 pH units upon metabolic inhibition (75/5/3; MWU, *** *p* < 0.001) (Figure 2c). Furthermore, the time to recover to baseline was significantly longer compared to WT (FWHM in NBCe1 KO: 513 ± 85 s; MWU, *** *p* < 0.001). In wild-type mice, chemical ischemia induced a [Na^+^]_i_ increase of 21.9 ± 7.2 mM (67/5/3). This [Na^+^]_i_ increase, from which cells fully recovered, was significantly smaller in NBCe1 KO astrocytes (16.2 ± 4.4 mM; 50/5/4; MWU, *** *p* < 0.001) (Figure 2d).

Taken together, these experiments show that pharmacological inhibition of NBC increases the amplitude of the ischemia-induced acidification in astrocytes while reducing the ischemia-induced [Na^+^]_i_ elevation. A similar result was obtained when comparing ion signals from astrocytes in slices from wild-type animals with those of NBCe1 KO animals. These data strongly suggest the activation of inwardly directed NBCe1 upon transient ischemia. While the resulting inward transport of HCO_3_^−^ dampens the astrocytic reduction in pH_i_, the accompanying Na^+^ influx increases their Na^+^ load.

### 3.3. Modeling Ion Dynamics and NBCe1 Activity during Transient Energy Deprivation

Based on the data on ischemia-induced changes in pH_i_ and [Na^+^]_i_ (see above), we aimed to develop a comprehensive model to simulate astrocytic NBCe1 activity during chemical ischemia. In addition to intracellular ion transients, chemical ischemia also depolarizes astrocytic membrane potential and changes extracellular ion concentrations, which will influence NBCe1 [10,41]. To make the simulation as realistic as possible, we additionally determined the most relevant of the latter parameters experimentally in our preparation.

**Figure 3:**
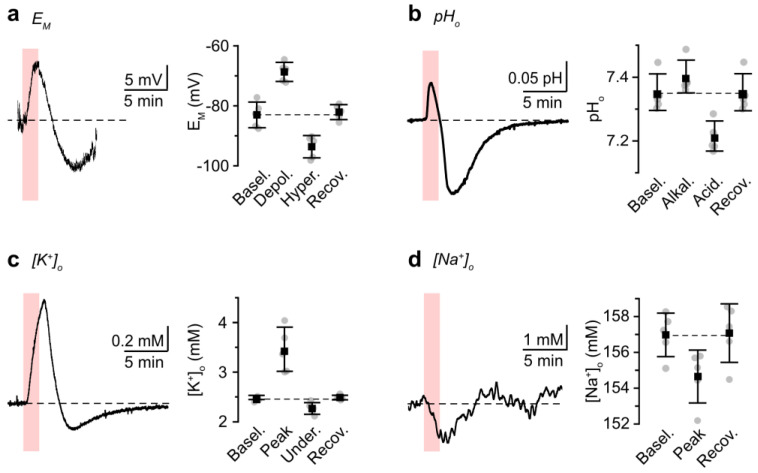
Ischemia-induced changes in astrocytic membrane potential and extracellular ions. (**a**) Change in astrocytic membrane potential (E_M_), measured in cell-attached mode, evoked by chemical ischemia for 2 min. (**b**) Change in pH_o_. Alkaline shifts are drawn upwards. (**c**,**d**) Change in [K^+^]_o_ and [Na^+^]_o_. Periods of chemical ischemia are indicated with the light-red areas. Dashed lines indicate baseline levels. Plots on the right summarize the results from these experiments and show individual data points (dots), means (squares), and SD (whiskers).

Astrocytic membrane potentials were measured in cell-attached mode to avoid artifacts induced via a dialysis of cells. Chemical ischemia for 2 min resulted in a transient depolarization of astrocytes by 14.3 ± 3.3 mV from a baseline resting membrane potential of −83.0 ± 4.3 mV. After the washout of the metabolic inhibitors, cells hyperpolarized by 10.5 ± 6.0 mV below the initial baseline, after which membrane potentials recovered (5/5/3) (Figure 3a). Extracellular ion concentrations were analyzed using ion-selective microelectrodes. Baseline pH_o_ was 7.35 ± 0.06. Chemical ischemia caused a biphasic alkaline-acid shift: pH_o_ first increased briefly by 0.05 ± 0.02 pH units, followed by a long-lasting drop to pH_o_ 7.22 ± 0.05, after which pH_o_ fully recovered (5/5/3) (Figure 3b). The baseline [K^+^]_o_ was 2.5 ± 0.0 mM. The induction of chemical ischemia caused a [K^+^]_o_ increase by 1.0 ± 0.5 mM, followed by an undershoot of 0.2 ± 0.1 mM below the initial baseline, from which [K^+^]_o_ slowly recovered towards the baseline (5/5/3) (Figure 3c). Finally, the baseline [Na^+^]_o_ was 156.3 ± 1.2 mM and reversibly decreased by 1.7 ± 1.0 mM upon chemical ischemia (5/5/3) (Figure 3d).

To incorporate the interaction of the modeled astrocyte with its environment (tissue and bath solution), the experimentally determined [Na^+^]_o_ and pH_o_ were fitted using polynomial functions of the form

(10) $$[{i]}_{bath}=[i]\prime_{bath}\sum_{j}p_{j}{\left(t-t_{0}\right)}^{n-j},$$

where i refers to Na^+^, K^+^, and pH; i′_bath_ represents the baseline concentration in the bath; *n* represents the degree of a polynomial; p represents its coefficient; j ranges from 0 to n; and t is the time. A polynomial of degree 9 was used for fitting [Na^+^]_bath_ and pH_bath_. Note that these functions representing concentrations or pH in the tissue are needed as the effects of other cells (neurons and other astrocytes) are not explicitly modeled and are different than the concentrations or pH immediately next to the cell. The concentrations or pH immediately next to the cell ([Na^+^]_o_, [K^+^]_o_, etc.) change dynamically in the model as the ions flow across the cell membrane (see Appendix A).

K^+^ in the bath ([K^+^]_bath_) is modeled using exponential functions to incorporate baseline, rise, decay, and recovery phases as

(11) $${\left[K^{+}\right]}_{bath}={\left[K^{+}\right]}_{bath}^{\prime }\;\;for\;\;t<t_{o,}\;-{\left[K^{+}\right]}_{bath}^{\prime }\left(\exp\left(k_{1}\left(t-t_{o}\right)\right)-k_{2}\right)\;\;for\;\;t>t_{0}\;\;and\;\;t<t_{0}+240,\;{\left[K^{+}\right]}_{bath}^{\prime }\left(\frac{\exp\left(k_{3}\left(-t_{0}-240\right)\right)}{k_{4}}+k_{5}\right)\;\;for\;\;t>t_{0}+240\;\;and\;\;t<t_{o}+535,\;-{\left[K^{+}\right]}_{bath}^{\prime }\left(\frac{\exp\left(k_{6}\left(t-t_{0}-535\right)\right)}{k_{7}}+k_{8}\right)\;\;for\;\;t>t_{0}+535$$

**Figure A1:**
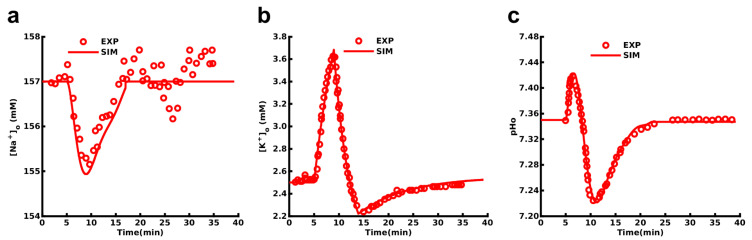
Fits to observed changes in [Na^+^]_o_ (**a**), [K^+^]_o_ (**b**), and pH_o_ (**c**). Solid lines and markers represent simulated and experimental values, respectively.

[K^+^]’_bath_ represents the baseline concentration of K^+^ in the bath. The fitting parameters k_1_, k_2_, k_3_, k_4_, k_5_, k_6_, k_7_, and k_8_ are used to model the rise, decay, and recovery phases, and their values are provided in Appendix A. Fits to the observed extracellular Na^+^, K^+^, and pH are shown in Figure A1.

**Figure 4:**
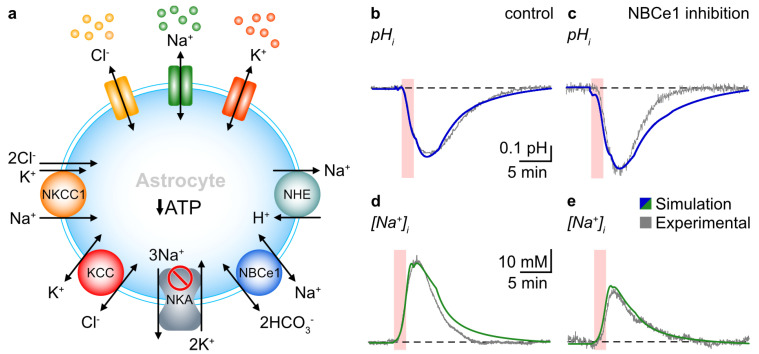
Computational modeling of ischemia-induced pH_i_ and [Na^+^]_i_ changes in astrocytes. (**a**) Schematic of the main pathways incorporated in the model. The arrow heads represent the direction of the flux. (**b**–**e**) Illustrating ischemia-induced pH_i_ and [Na^+^]_i_ changes in control (left panel) and upon inhibition of NBCe1 (right panel) in experimental and simulated conditions over a time course of 30 min. The blue and green traces represent simulated changes; the gray traces are experimental data taken from Figure 2a,b. Dashed lines indicate baseline levels. Periods of chemical ischemia are indicated with the light-red areas.

To model chemical ischemia, we decreased the activity of NKA by 2 min (see Equation (A4) in Appendix A). Furthermore, we assume that the local ischemia near the simulated astrocyte and recovery from ischemia set in slowly after the solution is switched from normal to chemical ischemia and vice versa. We mimicked this scenario in the model by decreasing and restoring the activity of NKA using sigmoidal functions (Appendix A). The activation of NBCe1 can lead to alterations in H^+^ and HCO_3_^−^ concentrations, resulting in changes in the pH_i_ of astrocytes in primary cell culture [57]. To simulate pH changes, we incorporated both NBCe1 and NHE into the model. In our simulations representing control mice, pH_i_ decreased from 7.33 to 6.92, while in simulations in which NBCe1 was inhibited, pH_i_ dropped to 6.77 (Figure 4b,c). These findings suggest that astrocytes in control conditions experience a smaller acidification in response to ischemia compared to cells lacking NBCe1. The smaller decrease in pH_i_ in cells expressing NBCe1 indicates that it acts as an acid extruder during chemical ischemia.

When chemical ischemia was active for 2 min, we also observed an increase in the [Na^+^]_i_. In control simulations, [Na^+^]_i_ increased by 34 mM from a baseline value of 12 mM (Figure 4d). However, in simulations mimicking an astrocyte with a 99% inhibition of NBCe1, [Na^+^]_i_ only rose by 26 mM (Figure 4e), indicating that NBCe1 activity is responsible for a 17% larger [Na^+^]_i_ increase. Notably, the results from these simulations are comparable to the experimental data, both concerning the kinetics and absolute amplitudes of the induced changes in pH_i_ and [Na^+^]_i_ (Figure 4).

### 3.4. NBCe1 Activity and ATP Consumption in Astrocytes

**Figure 5:**
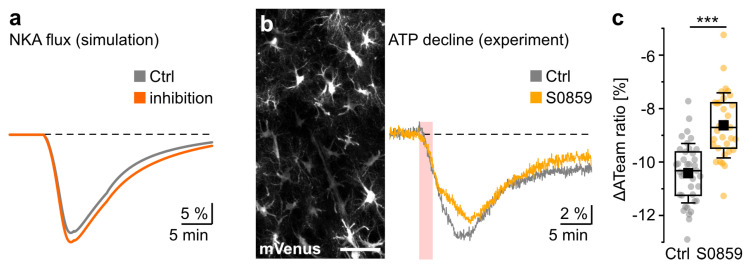
Role of NBCe1 in ischemia-induced astrocytic ATP depletion. (**a**) Computational modeling of reduction in the NKA flux in astrocytes. Gray represents the control flux, and light red represents the consumption rate upon inhibition of NBCe1. Note that NKA flux declines more with NBCe1 inhibition, indicating less NKA activity and, consequently, less consumption of ATP. (**b**) Left: Image of the ATeam fluorescence (mVenus) in cortical astrocytes. Scale: 50 µm. Right: Changes in the ATeam ratio during a 2 min induction of chemical ischemia in control and upon inhibition of NBC with S0859. Dashed lines indicate baseline levels. (**c**) Box plots summarizing the experimental results. Shown are individual data points (dots), means (squares), interquartile ranges (boxes), medians (lines), and SD (whiskers). *** *p* < 0.001. Periods of chemical ischemia are indicated with the light-red area.

Given the prominent role of NBCe1 in Na^+^ homeostasis, we next determined how inhibition of NBCe1 would affect ATP levels in astrocytes. We computed the consumption rate of ATP via NKA (knowing that NKA consumes one ATP molecule for transporting 3 Na^+^ out of the cell in exchange for 2 K^+^) by simulating the activity of NKA under 2 min of chemical ischemia in control and NBCe1-inhibited astrocytes. A comparison between the two conditions is shown in Figure 5a, where in both cases the NKA flux decreases (normalized to the resting state), reflecting the decreased activity of NKA and the resulting decline in its ATP consumption.

Interestingly, the NKA flux (and NKA’s ATP consumption rate) at the peak of chemical ischemia is lower in the astrocyte with NBCe1 inhibited compared to the control condition (Figure 5a). Thus, our model predicts that ATP levels should decline less during chemical ischemia in astrocytes with NBCe1 inhibited. Overall, this result suggests that NBCe1 activity promotes higher energy depletion during chemical ischemia.

To test the relation between NBCe1 and ATP depletion predicted using the model simulation, we conducted experiments in brain slices using FRET-based imaging with the ATP sensor ATeam1.03^YEMK^ (“ATeam”) expressed in astrocytes. Consistent with our earlier work [41,42], we observed a transient decrease in the ATeam ratio by 10.4 ± 1.1% in astrocytes subjected to chemical ischemia for 2 min (39/4/3) (Figure 5b,c), indicating a decrease in intracellular ATP. Within the observation period of 15 min after the washout of the blockers, cells partially recovered from this decline to an ATeam ratio that was about 4% lower than before chemical ischemia. During inhibition of NBC with S0859, chemical ischemia induced a decline in the astrocytic ATeam ratio by 8.6 ± 1.2%, which was significantly lower than in the control (33/3/3; ANOVA, *** *p* < 0.001) (Figure 5b,c). As observed in the control, ATP levels did not fully recover to their initial baseline values within the observation period of 15 min.

Taken together, both our simulation and experimental results indicate that inhibition of NBCe1 reduces the astrocytic decline in ATP induced via metabolic inhibition. Our data thus strongly suggest that activation of inward NBCe1 and the accompanying Na^+^ influx, respectively, promote energy depletion in ischemic conditions.

## 4. Discussion

In the present study, we demonstrate that a brief, 2 min period of chemical ischemia results in a decrease in the pH_i_ of astrocytes, which is accompanied by an increase in their [Na^+^]_i_. Pharmacological inhibition of NBC using S0859 caused a significant enlargement of the ischemia-induced astrocytic acidification and reduced the [Na^+^]_i_ elevation. Similar results were obtained when comparing ischemia-induced changes in astrocytic pH_i_ and [Na^+^]_i_ from wild-type animals with those of NBCe1 KO animals. Mathematical modeling based on our experimental data confirmed our experimental observations. Simulations furthermore predicted that activation of NBCe1 and the associated import of Na^+^ result in a higher flux of NKA and, thus, a higher cellular ATP consumption. This prediction was tested and confirmed experimentally via imaging cellular ATP, showing that NBCe1 promotes the loss of astrocytic ATP in ischemic conditions.

### 4.1. NBCe1 Activity Influences Astrocytic pH_i_ and [Na^+^]_i_

NBCe1 (*SLC4A4*) is highly expressed in astrocytes and is the major plasma membrane transporter responsible for cellular pH regulation at a pH_i_ above ~6.8 [19,20,21,22,58,59]. At more severe decreases in pH_i_; e.g., those accompanying severe ischemia, the Na^+/^H^+^ exchanger NHE1 is central for the export of protons [18,60,61]. The stoichiometry of NBCe1 in the forward mode is 1Na^+^:2HCO_3_^−^ [57,62,63], while in the reverse mode, it has recently been demonstrated to transport 1Na^+^:1HCO_3_^−^:1CO_3_^2−^ [64,65]. In physiological conditions, the reversal potential of NBCe1 is close to the typical resting membrane potential of forebrain astrocytes (about −85 mV; see, e.g., [41]). NBCe1 thus readily switches operating directions, e.g., following changes in the astrocytic membrane potential in pH_i_/[HCO_3_^−^]_i_ and/or in [Na^+^]_i_ [24,27,30,56].

Inward NBCe1 is accompanied by an increase in the [Na^+^]_i_ and an alkalinization of astrocytes [27,28,66]. This mode of operation is stimulated via neuronal activity and is a central element in neuro-metabolic coupling in the forebrain [23,25,26]. Moreover, inward NBCe1 is an integral part of the chemosensory Ca^2+^ signaling of astrocytes in the brainstem [67]. Inward NBCe1 is promoted with a K^+^-induced membrane depolarization of astrocytes, resulting in the so-called Depolarization-Induced Alkalinization (DIA; [50,51,62,68]). Reverse NBCe1, in contrast, serves the recovery from intracellular alkalosis [69] and may buffer extracellular acidifications [30]. It can be induced by switching from CO_2_/HCO_3_^−^-buffered to HEPES-buffered saline [27,69]. Reverse operation has also been shown upon inhibition of the NKA and the concomitant increase in astrocytic [Na^+^]_i_ [28].

In the present study, we probed for NBCe1 activity, employing two major strategies. One strategy was the comparison of intracellular pH and Na^+^ signals with and without the NBC blocker S0859. S0859 is an inhibitor of the NBC family and is not specific to NBCe1 [52]. Although it cannot be excluded that the effects obtained with S0859 might represent a mixture of inhibition of NBCe1 and other transporters such as NBCn1, it is well established that NBCe1 dominates in astrocytes [58]. In addition, S0859 has been reported to inhibit lactate transport via MCTs in astrocytes [70]. However, as the chemical ischemia protocol used in our study involves an inhibition of glycolysis, a significant production of lactate is unlikely under these conditions.

The other was a comparison of ion transients in preparations derived from wild-type mice with those from *Nbce1*-deficient mice. NBCe1 KO mice suffer from severe metabolic acidosis and other systemic effects and exhibit a sharp increase in mortality starting around postnatal day 8 [34,58]. To address the consequences of a deletion of NBCe1 on astrocytic ion changes, we therefore performed our study on organotypic tissue slice cultures prepared from neonatal animals. This is a well-established model system in which the cellular organization of neuronal networks is maintained and in which astrocytes continue to differentiate, similar to what is observed in vivo [71,72,73]. Notably, both strategies (application of S0589 to wild-type preparations as well as studying NBCe1 KO animals) produced similar results, indicating that S0859 indeed mainly targeted NBCe1 in astrocytes.

We first established that NBCe1 is functional in astrocytes in organotypic slices of the mouse neocortex by inducing a K^+^-induced DIA, which has been shown to involve NBCe1 activity [57,69]. This conclusion is based on the following observations: (1) brief elevation of [K^+^]_o_ from 2.5 to about 8.6 mM transiently increased astrocytic pH_i_; (2) the K^+^-induced alkalinization was sensitive to the NBC inhibitor S0859; (3) the amplitude of the K^+^-induced alkalinization was significantly smaller in slices prepared from animals deficient for NBCe1 compared to wild-type mice. (4) In addition to the described changes in pH_i_, the [K^+^]_o_ elevation resulted in a decrease in astrocytic [Na^+^]_i_. This phenomenon was reported before and can most likely be ascribed to an activation of the astrocytic NKA [2,13,27]. The K^+^-induced decrease in [Na^+^]_i_ was augmented in NBCe1 KO mice. The latter is indicative of a reduced Na^+^ influx in *Nbce1*-deficient mice, pointing to an NBCe1-mediated influx of [Na^+^]_i_ in wild-type animals. The results from our experiments thus confirm that NBCe1 activity results in detectable changes in the astrocytic pH_i_ and [Na^+^]_i_, thereby dynamically coupling intracellular Na^+^ homeostasis with cellular acid/base balance.

### 4.2. Operation of NBCe1 during Brief Chemical Ischemia

Brain ischemia results in a rapid decline in cellular ATP levels and in a reduction or failure of plasma membrane transporters, first and foremost of the NKA [7,74,75,76]. While cells of the ischemic core are doomed to die, the surrounding ischemic penumbra may recover if perfusion is restored in time. Recovery, however, is compromised with repeated waves of spreading depolarizations, which promote further damage [77]. Initially, spreading depolarizations are fully reversible and accompanied with a transient reduction in cellular ATP, an increase in [K^+^]_o_ and extracellular glutamate, as well as an increase in [Na^+^]_i_ and [Ca^2+^]_i_ and an intracellular acidification [9,10,11,32,78,79].

To mimic such conditions, we exposed tissue slices to blockers of cellular ATP production for 2 min (“chemical ischemia”). Earlier work has shown that chemical ischemia induces an immediate reduction in cellular ATP levels as well as changes in ion concentrations reminiscent of those observed during a spreading depolarization in the ischemic penumbra [10,41,42]. Here, we confirm these observations, demonstrating that brief periods of metabolic inhibition cause transient fluctuations in [K^+^]_o_, pH_o_, and [Na^+^]_o_, accompanied with a transient membrane depolarization, a decrease in pH_i_, and an increase in the [Na^+^]_i_ of astrocytes.

While results obtained in our study thus generally confirm earlier observations made using other in vitro models (oxygen–glucose deprivation or other strategies to mimic ischemic conditions; e.g., [7,10,55,80,81]) or using mouse models of ischemia in vivo [9,10,11,32,78,79], it is noteworthy that both the absolute amplitudes as well as the kinetics of ion changes can differ depending on the model employed. For example, it has been demonstrated earlier that the degree of extracellular acidification depends on the depth and duration of ischemia [80,82,83,84]. Compared to these studies, the pH_o_ changes determined in the present study were moderate. The same holds true for increases in [K^+^]_o_ determined here, which are lower in amplitude than those observed during spreading depolarizations in situ or in vivo [7,11]. A major reason for this discrepancy is the rather short duration of chemical ischemia employed. Moreover, brain slice preparations allow for more rapid extracellular diffusion and, thereby, may dampen extracellular ionic changes compared to the in vivo situation. In contrast, and as reported before [10], the amplitudes and time courses of [Na^+^]_i_ increases observed upon chemical ischemia were similar to those induced during a spreading depolarization in the ischemic penumbra of the mouse neocortex in vivo [10].

The cellular pathways of the ischemia-induced Na^+^ influx into astrocytes are still not fully understood. Notably, while moderate elevations in [K^+^]_o_ stimulate NKA and therefore cause a decrease in [Na^+^]_i_ (present work and [2,13,27]), spreading depolarizations and metabolic inhibition are additionally characterized by an increase in extracellular glutamate, as mentioned above. Indeed, blocking glutamate transporters strongly reduced Na^+^ increases in neocortical astrocytes exposed to transient energy depletion, suggesting a prominent Na^+^ influx into astrocytes via this pathway, temporarily overriding the capacity of NKA to export Na^+^ [10]. Inhibition of glutamate transporters reduced Na^+^ influx by only about 50%, implying that additional mechanisms for Na^+^ entry must exist. Our results obtained in the present study provide clear evidence for a stimulation of inward NBCe1 during chemical ischemia, showing that the latter represents such a pathway contributing to astrocytic Na^+^ loading. This conclusion is supported by the observation that blocking NBC with S0859 enlarged the acidification while at the same time reducing the [Na^+^]_i_ increase evoked by the metabolic inhibition. A similar observation was made using tissue from NBCe1 KO animals, in which the ischemia-induced acidification was enlarged and the ischemia-induced Na^+^ loading was reduced compared to wild-type animals. Stimulation of inward operation of NBCe1 in neocortical astrocytes upon brief chemical ischemia is in line with the proposed inward NBCe1 activity in cardiomyocytes upon myocardial ischemia [85] or in astrocytes exposed to long-lasting extracellular acidification to mimic cerebral ischemia [86].

Our experimental results are in line with the increased inward activity of NBCe1 transporters. As our experimental setting focused on the acute effects of brief metabolic inhibition (that is, on effects seen within a period of about 30 min), changes in expression levels are unlikely to play a role. Additionally, a substrate-mediated increase in transport activity, fast and efficient regulation of NBCe1 transport may occur via post-translational modifications such as phosphorylation or by changes in transporter trafficking to the plasma membrane, without changes in transcript or protein expression [29]. In the context of the present work, it is noteworthy that brain ischemia causes regulation of mTOR signaling (reviewed by [87]), which is known to be able to phosphorylate several residues of NBCe1, thereby altering its transport activity [88,89].

To further investigate the role of NBCe1 in astrocytic function, we integrated known physiological parameters and experimental data into a detailed biophysical model for ion concentrations and pH dynamics in the astrocyte and extracellular space. Our model predicted the inward activity of NBCe1 during chemical ischemia, which is consistent with our experimental findings. Notably, our modeling approach replicated increased inward NBCe1 activity in response to changes in ionic driving forces only, indicating that the latter will suffice to explain our experimental results. Furthermore, the model predicted that inhibition of NBCe1 activity results in a decrease in ATP consumption. This result suggests that the inward activity of NBCe1 exacerbates the depletion of ATP under metabolic stress. We validated these predictions in brain slices using FRET-based imaging with the ATP-sensor ATeam1.3^YEMK^. Overall, our mathematical model offers a complementary approach to exploring the complex dynamics of NBCe1-mediated Na^+^ and HCO_3_^−^ fluxes and how they modulate astrocytic [Na^+^]_i_, pH_i_, and ATP under different conditions.

### 4.3. Possible Consequences of NBCe1 Activity in the Ischemic Brain

The pathophysiological role of NBCe1 in the ischemic brain in vivo is still unclear. Several studies have reported an upregulation of sodium-bicarbonate cotransporters (NBC) in ischemic conditions or after induction of ischemic stroke in vivo. Jung et al. [90] showed significantly increased protein expression of NBC in the rat brain 3 and 6 h after a permanent middle cerebral artery occlusion (pMCAO) in rats. Moreover, Sohn and colleagues [91] demonstrated increased NBC protein levels in the hippocampal CA1 region of gerbils 12 h after exposure to transient cerebral ischemia, which, however, returned to control levels after 4 days. Astrocytes in culture, derived from human-induced pluripotent stem cells (hiPSCs), showed increased NBCe1 protein expression 24 h after exposure to a saline mimicking ischemic conditions in the brain [83].

Earlier work addressing the role of NBC transporters in brain injury concluded that their increased expression causes an increased vulnerability to extracellular acidosis by promoting the accumulation of intracellular Na^+^, thereby contributing to cellular damage in cerebral ischemia in vivo [86,90]. The study by Yao et al. [83], in contrast, showed that NBC activity protected astrocytes from cell death when exposed to ischemic conditions. Moreover, a recently posted manuscript [92] reports that loss of astrocytic NBCe1 increased the infarct volume after ischemic stroke in a mouse model in vivo. Results also indicate that NBCe1 dampens the breakdown of pH homeostasis in the ischemic brain [92].

The protective effect of NBCe1 might be related to its inward activity, increasing cell survival by reducing a harmful intracellular acidosis [18,86]. The latter idea is in line with the present study, in which we demonstrate that inward NBCe1 ameliorates the intracellular acidification in acute ischemic conditions. Intracellular acidosis is a harmful event, exacerbating cytotoxic edema and cellular damage through mechanisms including free radical generation, impaired protein synthesis, and/or intracellular Ca^2+^ accumulation [55,80,81,93,94,95]. Moreover, suppressing acidosis in astrocytes using optogenetic tools has been demonstrated to suppress glial glutamate release and ischemic brain damage in mouse models in vivo, presumably by reducing glutamate-induced excitotoxicity [96,97]. In another study, loss of NBCe1 was shown to cause familial hemiplegic migraine, and the authors suggested that a resulting lack of glial acid secretion into the ECS increased neuronal excitability [98].

The above-mentioned studies thus indicate that stimulation of inward NBCe1 and the resulting reduction in astrocytic acidosis and secretion of acid into the ECS, respectively, are neuroprotective. Its upregulation following ischemic stroke reported from in vivo mouse models [76,77] will support such a neuroprotective role. Regulation of pH, however, comes at a high price. Inward NBCe1 can drive water influx, astrocyte swelling, and shrinkage of the ECS [99,100,101,102], thereby exacerbating neuronal damage. Importantly, and as shown here, it also results in increased Na^+^ influx, aggravating the ischemia-induced Na^+^ loading of astrocytes. Besides weakening the driving force of Na^+^-dependent transporters, our present work demonstrates that NBCe1-related Na^+^ influx results in a larger decline in cellular ATP levels. The latter aggravates the loss of cellular energy upon chemical ischemia, most likely representing a harmful action of NBCe1, which might promote cellular damage in the ischemic brain. Clearly, further work, including studies in vivo, is needed to clarify the relevance of NBCe1 and its involvement in generating brain damage seen after stroke in animal models as well as in human patients.

## Data Availability

The experimental data are fully available from the authors (C.R.R./HHU) upon reasonable request. The codes reproducing the main results are available from the authors (G.U./USF) upon request.

## Appendix A. Model Equations and Parameters

The model used in this study builds on our previous work and uses the following equations [1]. K^+^ concentration in the ECS ([K^+^]_o_) depends upon the fluxes through Na^+^/K^+^−ATPase (J_NKA_), K^+^ channels (J_K_), Na^+^/K^+^/Cl^−^ co-transporter (J_NKCC1_), K^+^/Cl^−^ co-transporter (J_KCC1_), as well as K^+^ exchange with the bath solution (J_Kdiff_). The rate equation for [K^+^]_o_ is

(A1) $$\frac{{d[K^{+}]}_{o}}{dt}=\frac{1}{VR_{sa}}\left(J_{K}-2J_{NKA}-J_{NKCC1}-J_{KCC1}\right)+J_{Kdiff}$$

where VR_sa_ is the ratio of the ECS to the astrocytic volume. The flux through the K^+^ channels (μM s^−1^) is

(A2) $$J_{K}=G_{K}(v_{i}-E_{K})$$

where G_K_ is whole-cell conductance of K^+^ channels and v_i_ is the membrane potential. Strictly speaking, μM s^−1^ is not a standard flux unit; however, it can be converted to current density (pA/μm^2^) using a conversion factor of $10^{3}\frac{A}{F\times V}$, where *A*, *F*, and *V* is the area of the cell, Faraday’s constant, and volume of the cell, respectively. E_K_ is the reversal potential of K^+^ channels given using the Nernst equation (mV).

(A3) $$E_{K}=\frac{V_{T}}{z_{K}}\ln\left(\frac{[{K^{+}]}_{o}}{[{K^{+}]}_{i}}\right)$$

z_K_ is the valency of K^+^. J_NKA_ exports three Na^+^ and imports two K^+^. J_NKA_ (μM s^−1^) is given as

(A4) $$J_{NKA}=J_{NKA_{max}}(-I_{1}\left(a_{1},b_{1},t,t_{0},c_{1}\right)I_{2}\left(a_{2},b_{2},t,t_{0},c_{2}\right)\;\;\;\;\;\;\;\;\;\;\;\;\;\;\;\;\;\;\;\;\;\;\;\;\;\;\;\;\;\;\;\;\;\;\;\;\;\;\;\;\;+d)H_{1.5}\left([N{a^{+}]}_{i},K_{Na_{i}}\right)H\left({\left[K^{+}\right]}_{o},K_{K_{o}}\right)$$

where J_NKAmax_ is the maximum flux through Na^+^/K^+^−ATPase and H_n_(x,K) is from $\frac{x^{n}}{x^{n}+K^{n}}$, where n is the Hill coefficient, x is the concentration of Na^+^ or K^+^, and K in the function H_n_(x,K) is the dissociation constant of the respective ion to the pump.

(A5) $$I\left(a,b,t,t_{0},c\right)=\frac{a}{1+aexp\left(b\left(t-t_{0}\right)+c\right)}$$

where a, b, c, and d are constants, t represents time during the simulation and t_0_ represents the time at which ischemia is initiated. The sigmoidal forms are used to mimic the scenario where local ischemia near the cell settles in slowly or normal oxygen and glucose supply restore slowly after the solution is switched to ischemic condition and back to normal, respectively.

K^+^ diffusion between the ECS and bath solution (μM s^−1^) is given as

(A6) $$J_{Kdiff}=diff({\left[K^{+}\right]}_{bath}-{\left[K^{+}\right]}_{o})$$

where *diff* is the diffusion constant. One Na^+^, one K^+^, and two Cl^−^ ions move in inward direction through NKCC1. The flux through NKCC1 (μM s^−1^) is

(A7) $$J_{NKCC1}=G_{NKCC1}\emptyset \ln\left(\frac{[N{a^{+}]}_{o}[{K^{+}]}_{o}[C{l^{-}]}_{o}^{2}}{[N{a^{+}]}_{o}{\left[K^{+}\right]}_{i}[C{l^{-}]}_{i}^{2}}\right)$$

G_NKCC_ is the whole-cell conductance of NKCC1. Cl^−^ and K^+^ flux through KCC1 channels (μM s^−1^) is

(A8) $$J_{KCC1}=G_{KCC1}\emptyset \ln\left(\frac{{\left[K^{+}\right]}_{o}[C{l^{-}]}_{o}}{[{K^{+}]}_{i}[C{l^{-}]}_{i}}\right),$$

where G_KCC1_ is the whole-cell conductance of KCC1.

The rate equation for [Na^+^]_o_ depends on the flux through Na^+^ channels (J_Na_), NKA, NBCe1, Na^+^/H^+^ exchanger, and Na^+^ exchange with the bath solution (J_Nadiff_).

(A9) $$\frac{d[N{a^{+}]}_{o}}{dt}=\frac{1}{VR_{sa}}\left(J_{Na}+3J_{NKA}-J_{NKCC1}-J_{NBCe1}+J_{NHE}\right)+J_{Nadiff},$$

Na^+^ flux through Na^+^ channels (μM s^−1^) is

(A10) $$J_{Na}=G_{Na}(v_{i}-E_{Na})$$

where G_Na_ is the whole-cell conductance of Na^+^ channels and E_Na_ is the reversal potential for Na^+^.

(A11) $$E_{Na}=\frac{V_{T}}{z_{Na}}\ln\left(\frac{{\left[{Na}^{+}\right]}_{o}}{{\left[Na^{+}\right]}_{i}}\right)$$

Fluxes through NBCe1 and NHE (μM s^−1^) are given in Section 2.6.

Na^+^ exchange with the bath solution (μM s^−1^) is given as

(A12) $$J_{Nadiff}=diff({\left[{Na}^{+}\right]}_{bath}-{\left[Na^{+}\right]}_{o})$$

where diff is the diffusion constant of Na^+^.

K^+^ concentration in the astrocyte ([K^+^]_i_) depends on the fluxes due to K^+^ channels, NKA, NKCC1, and KCC1. That is,

(A13) $$\frac{{d[K^{+}]}_{i}}{dt}=-J_{K}+2J_{NKA}+J_{NKCC1}+J_{KCC1}.$$

[Na^+^]_i_ depends on the fluxes through Na^+^ channels, NKA, NBCe1, and NHE. These fluxes are already described above.

(A14) $$\frac{{d[Na^{+}]}_{i}}{dt}=-J_{Na}-3J_{NKA}+J_{NKCC1}+J_{NBCe1}-J_{NHE}$$

Cl^−^ concentration in the astrocyte ([Cl^−^]_i_) and ECS ([Cl^−^]_o_) is given via electroneutrality (μM).

(A15) $$\frac{d[C{l^{-}]}_{i}}{dt}=\frac{d[N{a^{+}]}_{i}}{dt}+\frac{d{\left[K^{+}\right]}_{i}}{dt}-J_{NBCe1}$$

(A16) $$[C{l^{-}]}_{o}=[N{a^{+}]}_{o}+[{K^{+}]}_{o}-{\left[HCO_{3}^{-}\right]}_{o}$$

Membrane potential of the astrocyte (*mV*) is given as

(A17) $$\frac{dv_{i}}{dt}=\gamma_{v}(-J_{K}-J_{Na}-J_{Cl}-J_{NKA}+J_{NBCe1}-J_{NHE})$$

where γ_v_ converts flux from concentration unit to current unit. Cl^−^ flux through leak channels (μM s^−1^) is

(A18) $$J_{Cl}=G_{Cl}(v_{i}-E_{Cl})$$

where G_Cl_ is the maximum conductance of Cl^−^ channels. E_Cl_ is the reversal potential of Cl^−^ and is given using

(A19) $$E_{Cl}=\frac{v_{T}}{z_{Cl}}\ln\left(\frac{{\left[Cl^{-}\right]}_{o}}{{\left[Cl^{-}\right]}_{i}}\right)$$

where z_Cl_ is the valence of Cl^−^.

The parameters used in the model are given in Table A1, Table A2, Table A3 and Table A4 (see below).

**Table A1:** Parameters used in the equations for ion dynamics in the astrocyte and ECS as well as membrane potential of the astrocyte.

Parameter	Description	Value
γ_v_	Scaling factor for membrane potential	1970 mV μM^−1^
VR_sa_	Volume ratio between the ECS and astrocyte	3
V_T_	Voltage constant in Nernst equation	26.7 mV
G_K_	Peak conductance of K^+^ channels	2072.3 μM mV^−1^ s^−1^
G_Na_	Peak conductance of Na^+^ channels	68.08 μM mV^−1^ s^−1^
G_NBCe1_	Peak conductance of NBCe1	392.22 μM mV^−1^ s^−1^
G_NHE_	Peak conductance of NHE	$\frac{1}{3}$G_NBCe1_
J_NaKmax_	Maximum flux through NKA	4.26 × 10^4^ µM s^−1^
K_Na_	Association constant for Na^+^ to NKA	10 × 10^3^ µM
K_Ko_	Association constant for K^+^ to NKA	1.5 × 10^3^ µM
diff	Diffusion constant of Na^+^, K^+^, and pH	0.5 s^−1^
β_i_	Intracellular intrinsic buffering capacity	25 mM/pH unit
β_o_	Extracellular intrinsic buffering capacity	10.5 mM/pH unit
pK_a_	Negative logarithm of the acid dissociation constant of carbonic acid	6.1
s	Solubility of CO_2_	2.25 × 10^−4^ mM Pa^−1^
P_CO2_	Partial pressure of CO_2_	5332.9 Pa
K_h_	Dissociation constant of CO_2_	800 nmol L^−1^

**Table A2:** The initial values of state variables.

Parameter	Description	Value
[Na^+^]_o_	Extracellular Na^+^ concentration	157 mM
[Na^+^]_i_	Intracellular Na^+^ concentration	12 mM
[K^+^]_o_	Extracellular K^+^ concentration	2.5 mM
[K^+^]_i_	Intracellular K^+^ concentration	146 mM
V_i_	Intracellular membrane potential	−83 mV
pH_o_	Extracellular pH value	7.35
pH_i_	Intracellular pH value	7.32

**Table A3:** Parameters used in functional forms of ions in the bath.

Parameter	Description	Value
${\left[K^{+}\right]}_{bath}^{\prime }$	Baseline value of [K^+^]_bath_	2.5 mM
	k_1_	−0.002675 s^−1^
	k_2_	−2
	k_3_	−0.0052 s^−1^
	k_4_	1.350
	k_5_	0.73
	k_6_	−0.00169
	k_7_	7.6
	k_8_	−1.02
$[{Na^{+}]}_{bath}^{\prime }$	Baseline value of [Na^+^]_bath_	157 mM
	p_1_	−1.1906 × 10^−26^ s^−1^
	p_2_	5.045 × 10^−23^ s^−1^
	p_3_	−8.122 × 10^−20^ s^−1^
	p_4_	5.6637 × 10^−17^ s^−1^
	p_5_	−7.006 × 10^−15^ s^−1^
	p_6_	−1.3156 × 10^−11^ s^−1^
	p_7_	7.521 × 10^−9^ s^−1^
	p_8_	−1.326 × 10^−6^ s^−1^
	p_9_	3.51 × 10^−6^ s^−1^
	p_10_	1.0005
${pH}_{bath}^{\prime }$	Baseline value of $pH_{bath}$	7.35 − 5.4275 × 10^−19^ s^−1^
	p_1_	−4.7273 × 10^−26^ s^−1^
	p_2_	2.47264 × 10^−22^ s^−1^
	p_3_	−5.4275 × 10^−19^ s^−1^
	p_4_	6.4515 × 10^−16^ s^−1^
	p_5_	−4.4486 × 10^−13^ s^−1^
	p_6_	1/7487 s^−10^
	p_7_	−3.4571 s^−8^
	p_8_	2.0687 × 10^−6^ s^−1^
	p_9_	1.0069 × 10^−4^ s^−1^
	p_10_	0.9999

**Table A4:** Parameters used in the NKA flux.

Parameter	Description	Value
a_1_	Parameter in sigmoidal function	1000
b_1_	Parameter in sigmoidal function	0.0022134 s^−1^
c_1_	Parameter in sigmoidal function	0
t_0_	Beginning of pump inhibition	300 s
a_2_	Parameter in sigmoidal function	1.52
b_2_	Parameter in sigmoidal function	−0.02014 s^−1^
c_2_	Parameter in sigmoidal function	5.738
_d_	Intercept parameter in sigmoidal functions	1.13
